# Challenging inferior vena cava filter removal complicated by strut penetration and prolonged dwell

**DOI:** 10.1016/j.jvscit.2025.101894

**Published:** 2025-06-21

**Authors:** Valentyna Kostiuk, Paula Pinto Rodriguez, Britt H. Tonnessen, Juan Carlos Perez Lozada, Raul J. Guzman, Cassius Iyad Ochoa Chaar

**Affiliations:** a Yale School of Medicine, New Haven, CT; b Division of Vascular Surgery and Endovascular Therapy, Department of Surgery, Yale School of Medicine, New Haven, CT; c Section of Vascular & Interventional Radiology, Department of Radiology & Biomedical Imaging, Yale School of Medicine, New Haven, CT

**Keywords:** Inferior vena cava filter, Inferior vena cava, Venous thromboembolism, Wire loop technique

## Abstract

Temporary placement of inferior vena cava (IVC) filters for pulmonary embolism prevention is indicated for patients with contraindications to or unsuccessful anticoagulation therapy. However, timely IVC filter removal is necessary to avoid common complications, including filter strut penetration through the IVC wall into surrounding organs and structures. This report and describes IVC filter removal in a patient with symptomatic strut erosion into the L4 vertebral body and abutment of the proximal right common iliac artery, which was performed 12 years after placement. After filter retrieval, the patient's symptoms resolved, and he remains stable on anticoagulation therapy.

Retrievable inferior vena cava (IVC) filters effectively reduce the risk of pulmonary embolism, particularly in patients with venous thromboembolism who cannot undergo or have not responded to anticoagulation therapy.^1^ However, prolonged dwell time can cause complications, making timely filter retrieval essential once it is no longer needed.^2^ However, filter retrieval rates remain low, particularly among Medicare patients, at just 18%.^3^ Struts of retained IVC filters may penetrate adjacent structures, including the aorta, duodenum, renal artery, and vertebral bodies.^4^^,^^5^ Approximately 19% of patients with IVC filters experience IVC wall penetration, with 8% of patients reporting significant discomfort, most commonly manifesting as pain.^6^ Beside prolonged dwell time, lateral tilt has also been associated with complications, the need for advanced filter retrieval techniques, and the development of new retrieval devices (Supplementary Video, online only).^7^^,^^8^ This report describes a variation of the wire loop technique with double snaring using a trilobe snare for retrieving a tilted IVC filter with dwell time of 12 years in a patient who presented with back pain from strut penetration into the spine (Supplementary Video, online only). The patient provided informed consent for this publication.

## Case report

**Fig 1:**
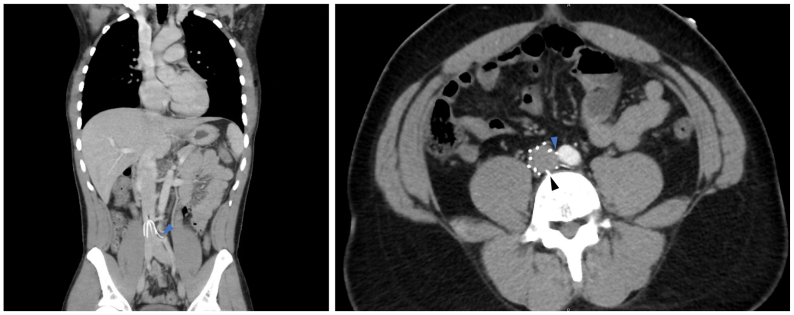
Preoperative computed tomography (CT) scan showing the inferior vena cava (IVC) filter struts eroding into the L4 vertebral body (black arrowhead) and abutting the proximal right common iliac artery (blue arrowhead).

A 27-year-old male patient without a significant past medical history presented for an IVC filter retrieval. Twelve years prior, he had bilateral femoral vein deep venous thromboses (DVTs) after multiple gunshot wounds. At the time of injury, the patient underwent left superficial femoral artery repair using an 8-mm Gore-Tex interposition graft (W. L. Gore & Associates, Newark, DE) with bilateral superficial femoral vein ligation and bilateral four-compartment fasciotomy. A Bard Eclipse IVC filter (Bard Peripheral Vascular, Tempe, AZ) was placed in the infrarenal position. Six months earlier, the patient was involved in a car accident, and a computed tomography scan showed the IVC filter's struts eroding into the L4 vertebral body, while also abutting the right common iliac artery (Fig 1, Supplementary Video, online only). The patient reported positional lower back pain after a recent accident. He denied having back pain before the accident and IVC filter placement. He had no recent DVTs or calf pain. Bilateral lower extremity venous duplex ultrasound examination confirmed the absence of DVT, and IVC filter removal was performed.

**Fig 2:**
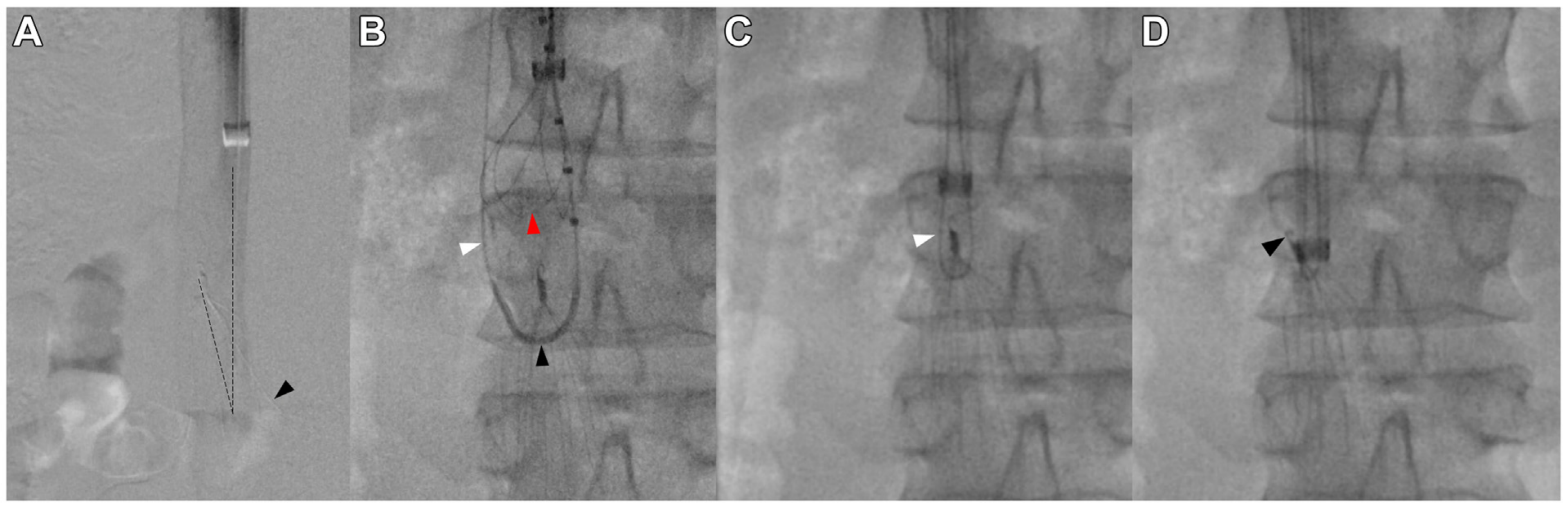
**(A)** A venogram showing a 9° tilted inferior vena cava (IVC) filter (dashed lines outlining the IVC and the filter axes) as well as the penetration of the IVC filter strut outside of the IVC wall (black arrowhead). **(B)** An Omniflush catheter (black arrowhead) positioned across the base of the filter to direct the Glidewire (white arrowhead) to be captured by a trilobe snare (red arrowhead). **(C)** A backward pressure is applied on the wire loop (white arrowhead) to align the sheath and the IVC filter hook; the sheath can be then advanced to capture the filter. **(D)** A misalignment of the IVC filter hook and the sheath demonstrated by the location of the hook outside of the sheath (black arrowhead).

**Fig 3:**
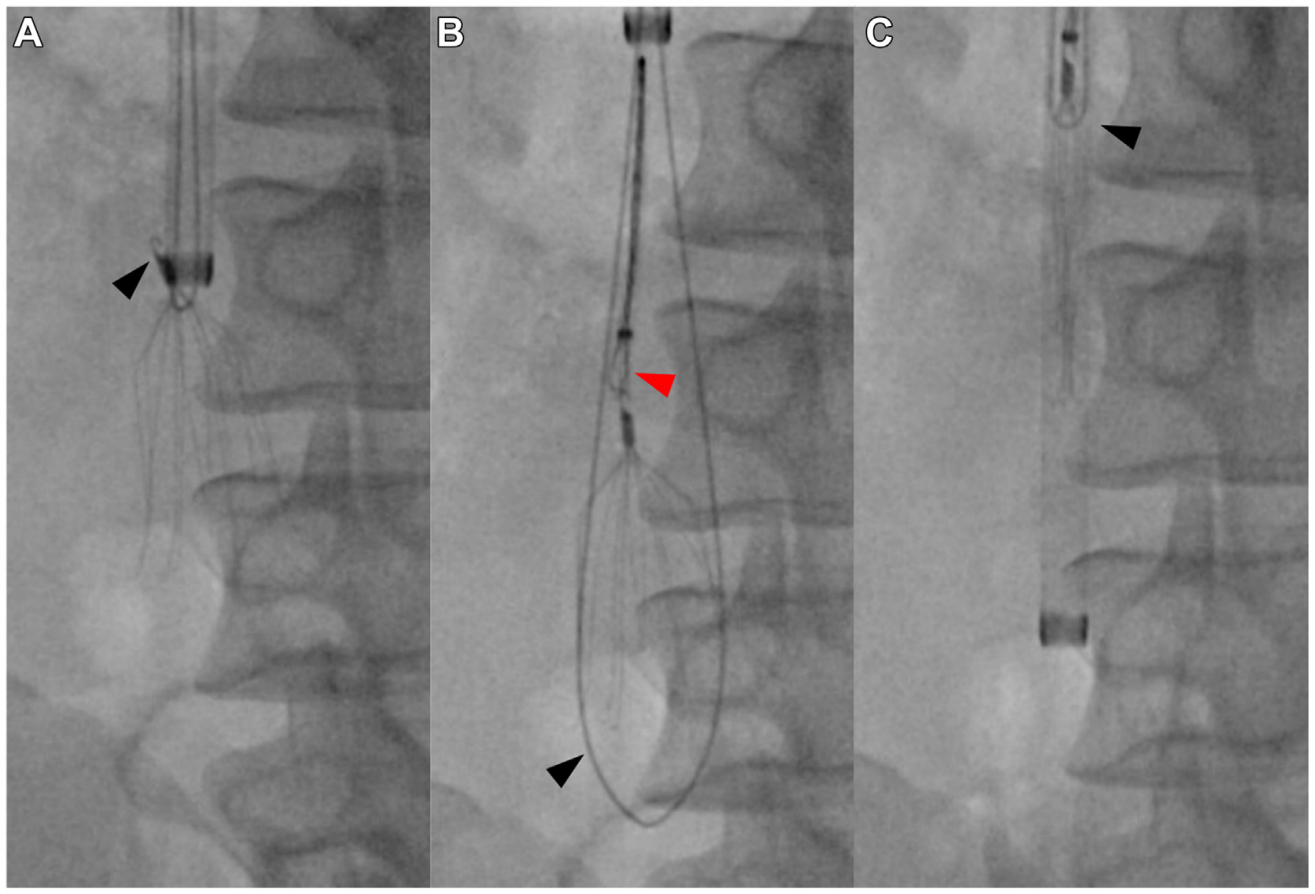
**(A)** Increased magnification and different projection angles demonstrated misalignment between the sheath and the filter hook (black arrowhead). **(B)** Moving the sheath and the wire loop (black arrowhead) further away from the inferior vena cava (IVC) filter allowed to expand the snare (red arrowhead) and capture the filter hook. **(C)** As the sheath was advanced, the IVC filter (black arrowhead) was collapsed and retrieved.

The right neck and bilateral groins were prepped and draped ;femoral access is sometimes needed in challenging IVC filter retrievals. A venogram showed a 9° lateral filter tilt without thrombus (Fig 2, *A*). An advanced wire loop technique was chosen because it provides a sturdy handle on the filter neck as opposed to snaring the hook only. After a 16F sheath (Cook Medical, Bloomington, IN) was introduced, an Omniflush catheter (AngioDynamics, Inc., Latham, NY) was advanced across the filter base to direct a 0.035-in soft Glidewire (Terumo, Somerset, NJ) across the filter base spanning across the hook (Fig 2, *B*). Passing a wire loop across the filter struts only without engaging the filter neck should be avoided because it can cause strut fracture. With the wire being advanced, a 20- or 30-mm trilobe ENsnare (Merit Medical Systems, Inc., South Jordan, UT) was introduced through the sheath in a buddy catheter fashion. Once captured by the snare, the wire is exteriorized and a snap is placed across both ends of the wire close to the sheath to be used as a handle. The sheath was then advanced with gentle backward tension kept on the wire loop to optimize the alignment of the IVC filter tip and the sheath (Fig 2, *C*). As the sheath was advanced to capture the filter hook, some resistance was encountered. Increased magnification and different projection angles demonstrated that the sheath did not engage the filter hook (Figs 2, *D*, and 3, *A*). The sheath was then pulled back and manipulated outside of the patient's body to change its angle and configuration. Despite this maneuver, the sheath did not capture the filter hook (Fig 3, *A*). The snare was then reintroduced to capture the filter hook. Initial snaring attempts were unsuccessful because the wire loop and the sheath prevented full extension of the snare around the filter hook. At that point, the sheath and the wire loop were moved further away from the filter, and the hook was finally captured by the snare (Fig 3, *B*). Next, the sheath was advanced with gentle tension applied to the wire loop and the snare simultaneously, ensuring proper alignment and capture of the filter hook (Fig 3, *C*). The completion angiogram showed excellent flow without dissection, thrombus, perforation, or extravasation. After filter removal, the patient's back pain had resolved. Two years after filter removal, the patient was found to have a left femoral vein thrombosis and was started on 5 mg apixaban (Eliquis) twice daily. He underwent magnetic resonance angiography of the abdomen and pelvis, which demonstrated patent vessels without central venous stenosis or thrombosis.

## Discussion

Penetration of the struct of an IVC filter into the vertebral body is rare, with an estimated incidence of 0.7%.^6^ Approximately 30% of patients who develop this complication are symptomatic, with back pain being the most common complaint.^6^ In this patient, the advanced wire loop technique was necessary to remove an IVC filter owing to a prolonged dwell time of 12 years and vertebral body penetration. Although there are no reports of IVC filter removal involving both a similar prolonged dwell time and spinal penetration, one report detailed successful IVC filter retrievals from two patients with vertebral body penetration after a dwell time of approximately 1 year.^5^ The first patient underwent filter removal 1 year after placement. Similar to the current patient, preprocedure computed tomography showed that the filter significantly tilted toward the anterior IVC wall, with one of the struts penetrating the L4 vertebral body. Using right internal jugular vein access, a modified loop snare technique was used to detach the hook embedded in the IVC wall. The hook was engaged with an ENsnare, and the filter was collapsed and removed by advancing the sheath. The second patient in this series had an IVC filter placed 8 months earlier for an acute DVT, and the IVC filter was removed successfully using the ENsnare device only. The authors emphasized that, in both cases, the filter was removed without any excessive tension on the system, which was likely owing to the relatively short dwell times. In contrast, gentle tension was applied using both a wire loop and a snare to remove the IVC filter in the current patient; the prolonged dwell time most likely contributed to scar tissue formation around the filter struts.

In another report, a 24-year-old woman underwent placement of a Günther Tulip filter (Cook Medical, Bloomington, IN) in an infrarenal position in the setting of disseminated intravascular coagulation owing to preeclampsia and DVT.^9^ Two years later, she presented with back pain and was found to have a fractured filter strut embedded in the vertebral body. A venogram performed during the retrieval procedure showed hook apposition to the IVC wall. A guidewire loop technique was used to separate the hook from the IVC wall and remove the fractured filter. Although the fractured strut remained embedded in the vertebral body, the patient reported no recurrent symptoms with complete resolution of back pain.

## Conclusions

This report describes the treatment of a patient with symptomatic IVC filter strut penetration into the L4 vertebral body. An advanced wire loop technique with double snaring was used successfully to remove the filter 12 years after initial placement.

## Funding

None.

## Disclosures

None.

## Appendix (online only)

Supplementary Video (online only) Case presentation and technical aspects of an advanced wire-loop technique for inferior vena cava (IVC) filter removal, along with the testing of the next-generation retrieval device that could improve complex retrieval procedures.
